# Effect of Microbial Phytase on Ileal Digestibility of Minerals, Plasma and Urine Metabolites, and Bone Mineral Concentrations in Growing–Finishing Pigs

**DOI:** 10.3390/ani12101294

**Published:** 2022-05-18

**Authors:** Anna Czech, Wioletta Samolińska, Ewa Tomaszewska, Siemowit Muszyński, Eugeniusz R. Grela

**Affiliations:** 1 Department of Biochemistry and Toxicology, Faculty of Animal Sciences and Bioeconomy, University of Life Sciences in Lublin, 12 Akademicka Street, 20-950 Lublin, Poland; anna.czech@up.lublin.pl; 2 Institute of Animal Nutrition and Bromatology, Faculty of Animal Sciences and Bioeconomy, University of Life Sciences in Lublin, 13 Akademicka Street, 20-950 Lublin, Poland; eugeniusz.grela@up.lublin.pl; 3 Department of Animal Physiology, Faculty of Veterinary Medicine, University of Life Sciences in Lublin, 12 Akademicka Street, 20-950 Lublin, Poland; ewarst@interia.pl; 4 Department of Biophysics, Faculty of Environmental Biology, University of Life Sciences in Lublin, 13 Akademicka Street, 20-950 Lublin, Poland; siemowit.muszynski@up.lublin.pl

**Keywords:** phytase, pigs, elements, intestines, excreta, bone

## Abstract

**Simple Summary:**

In the presented study, increasing phytase activity in the diet of grower–finisher pigs improved the bioavailability of minerals, increased their concentration in the femurs, and reduced their fecal and urinary excretion. The results of the research provide new information on the positive effect of the use of an appropriate level of microbial phytase supplementation, which reduces the anti-nutritional effect of phytates by improving the availability of minerals (mainly P, Ca, but also Mg, Zn, Cu, and Fe) for the animal, which, at the same time, limits the negative impact of unused minerals on the natural environment.

**Abstract:**

The study was conducted to evaluate the effects of added phytase in the diet of pigs on utilization of minerals and to determine the essential levels of this supplement in compound feed. An additional aim of the study was a critical assessment of current recommendations regarding the level of phosphorus in the diet of pigs, taking into account the use of phytase. A total of 432 pigs were allotted to six dietary treatments, with nine replicate pens per treatment according to body weight (BW) and sex. The treatments included a negative control (NC) with reduced content of digestible phosphorus; the NC diet supplemented with 6-phytase produced by a genetically modified strain of *Aspergillus oryzae* in the amount of 250 (NC + 250), 500 (NC + 500), 1000 (NC + 1000), or 1500 (NC + 1500) FTU/kg of feed; and a positive control (PC) diet formulated to meet NCR nutrient requirements for pigs. The results showed that, the higher the phytase activity in the diet (NC+), the lower the concentration of P, Ca, and Mg in the chyme (*p* < 0.05). Pigs fed the PC and NC+ diets had higher plasma levels of P and Ca than group NC in both fattening periods (*p* < 0.05). The content of phosphorus in the femur of pigs increased with the level of phytase added to the diet (*p* < 0.05). The content of Cu and Zn in the femur of pigs in the growing period was higher in groups NC + 500 and NC + 1000 than in the remaining experimental groups (*p* < 0.001). The content of P and Ca in the feces was higher in pigs fed the PC diet in comparison to the remaining experimental groups in both fattening periods (*p* < 0.001). There was a decrease in the content of P, Ca, and Mg in the excreta of pigs fed NC+ diets in both fattening periods (*p* < 0.05). A linear decrease in excretion of zinc in the feces was noted in the case of high levels of phytase, i.e., 1000 and 1500 FTU (*p* < 0.001). Increasing the level of phytase decreased the Cu (*p* < 0.001) content in the urine of growing–finishing pigs. In conclusion, the analysis of the effects of the use of phytase in a range of 0–1500 FTU/kg in low-phosphorus diets for fattening pigs indicates that 1000 FTU is the most effective level of phytase for increasing utilization of minerals and reducing excretion of elements into the environment.

## 1. Introduction

Due to the low activity of endogenous phytase and limited microbial population in the small intestine of monogastric animals, their gastrointestinal tract has limited ability to hydrolyze phytate, meaning that phosphorus bound to phytate is of low bioavailability [1,2]. About two-thirds of phosphorus of plant origin is believed to occur in the form of phytic acid, i.e., in the form of myo-inositol phosphates of very low bioavailability [1]. Phytates not only limit the availability of phosphorus, but also, by forming complexes with calcium and other divalent elements, reduce the bioavailability of other minerals important for systemic homeostasis. In the neutral pH environment of the gastrointestinal tract, carboxyl groups of amino acids can bind phytates through divalent cations (Ca^2+^, Mg^2+^, Zn^2+^, Cu^2+^, Fe^2+^, Mn^2+^, Mo^2+^, and Co^2+^). This can cause disturbances in mineral metabolism and thereby impair bone mineralization, leading to osteomalacia or susceptibility to excessive bone fragility, as well as increased embryo mortality. Furthermore, in the acidic environment of the stomach (pH < 4.5), phytates also form complexes with proteins, which negatively affects the availability and digestibility of amino acids [3].

Interest in phytase is also rising as a result of the need to reduce the use of inorganic phosphates in compound feed for pigs, due to high production costs (phosphorus is believed to be the third most expensive nutrient, after energy and protein) [4] and concerns about the effects of animal production on the environment. Phytate phosphorus, which is unavailable for monogastric animals, is excreted and contributes to environmental pollution, especially in areas with a high density of animal production.

Research has shown that the addition of phytase to the diet of pigs (especially piglets and pregnant and lactating sows) significantly increases the bioavailability of minerals, resulting in a marked decrease in their excretion in the feces and urine [1,5]. By increasing the bioavailability of calcium (32%), magnesium (8–13%), zinc (7–13%), copper (3–7%), and iron (2–9%) in diets for pigs, the amounts of these elements added to feed can be reduced by at least 20% [6].

Higher bioavailability of minerals from compound feed increases the efficiency of absorption from the intestine and transport into the bloodstream. This allows many biochemical processes to proceed normally and helps to maintain overall homeostasis in the body, including mineral homeostasis. Improvement in the bioavailability of minerals is reflected in their increased levels in the colostrum and milk [7], as well as in improved hematological parameters of the blood [8]. An analysis of the bones of monogastric animals receiving feed supplemented with microbial phytase indicated a positive effect of this enzyme on bone mineralization [9].

Although the benefits of exogenous phytase in the diet of pigs and poultry are well-known [10], research is still being carried out on phytase obtained from new strains of microbes. Phytases from *Escherichia coli* provide greater benefits than fungal phytase in terms of nutrient digestibility and reduced excretion of phosphorus (P) in broiler chickens [11] and pigs [12]. A better understanding of the mechanisms of utilization and distribution of P, Ca, Mg, Zn, Fe, and Cu in conditions of phytase supplementation in the diet can help to maximize the benefits of phytase resulting from the increased availability of many minerals and the associated reduction in environmental pollution.

While the effects of microbial phytase are well documented, it should be noted that phytases are constantly evolving in terms of how effectively they improve the bioavailability of mineral compounds from feed. Only a few types of phytase have been described as highly specific for phytic acid. These include phytases from *Bacillus* spp., *Aspergillus* spp., and *E. coli*, and those belonging to the class of PTP-phytases. The action of these phytases involves hydrolysis of phytin (IP6—six phosphate groups) via gradual dephosphorylation to IP5, IP4, IP3, IP2, and IP1. A preparation of 6-phytase produced by a genetically modified strain of *Aspergillus oryzae* (DSM 22594) is highly specific for phytic acid. This phytase is highly stable in gastrointestinal conditions (in vitro test) and is more resistant to feed production conditions, particularly pelleting. The very promising improvements shown in fattening efficiency and general bone mineralization [9] prompted us to broaden the scope of research on the effectiveness of this type of phytase used as a diet supplement, primarily its effect on the bioavailability and utilization of minerals.

Therefore, the aim of the study was to determine the effect of added phytase produced by a genetically modified strain of *Aspergillus oryzae* (DSM 22594) in the diet of pigs on utilization of minerals and to determine the essential levels of this supplement in compound feed. The study also includes a critical assessment of current recommendations regarding the level of phosphorus in the diet of pigs, taking into account the use of phytase.

## 2. Materials and Methods

The experimental protocol and all experimental procedures used in this study were approved by the Second Local Ethics Committee on Animal Experimentation of the University of Life Sciences in Lublin, Poland.

### 2.1. Animals

A total of 432 pigs were used in the study: 216 barrows and 216 gilts, progeny of Large White Polish x Polish Landrace crossbreds. The pigs, obtained from a local breeder practicing conventional commercial farming, arrived at the experimental facility with body weight (BW) of ~35 kg. On the day of arrival, the pigs were weighed individually, ear-tagged, and allocated at random to dietary treatment groups of 72 animals each, with barrows and gilts in equal numbers. Each dietary treatment consisted of nine replicate straw-bedded pens with 8 pigs per pen, balanced for sex distribution (4 gilts + 4 barrows) and minimalized within the variation of body weight as far as practically possible.

### 2.2. Experimental Design

The treatment groups included a positive control (PC) group fed a diet containing calcium and phosphorus levels that met NCR nutrient requirements for growing (6.62 g/kg and 5.69, respectively) and finishing (6.09 g/kg and 5.27, respectively) pigs [13]; a negative control (NC) group whose diet had reduced content of calcium (83% and 85% of PC diet for grower and finisher, respectively) and phosphorus (85% and 75% of PC diet for grower and finisher, respectively); and four experimental groups fed diets similar to the NC diet but with the addition of 250, 500, 1000, or 1500 phytase units (FTU)/kg of 6-phytase produced by a genetically modified strain of *Aspergillus oryzae* (RONOZYME HiPhos, DSM Nutritional Products, Mszczonów, Poland) with declared minimal activity of 50,000 FTU/g. Phytase was added to the phytase-supplemented diets as a premix. Within each treatment, pigs were fed diets that were appropriate to the stage of production, i.e., grower (35–70 kg average BW) and finisher (70–110 kg average BW). The composition of the diets has been described previously [9] (Table 1). All experimental diets were prepared and pelleted by DSM Poland. The animals had ad libitum access to feed and water throughout the experiment.

### 2.3. Feed Analysis

Feed samples were analyzed for dry matter and crude ash content and for total P, Ca, Mg, Cu, Fe, and Zn concentrations, using standard AOAC [14] methods [9] (Table 1). Total phytase activity was quantified by a colorimetric enzymatic method [14]. One FTU was defined as the amount of enzyme required to release 1 μmol of inorganic phosphorus per minute from sodium phytate at 37 °C [15]. In all diets, the determined phytase activity was within the target range or slightly higher [9] (Table 1).

### 2.4. Sampling and Measurements

Blood for analysis was sampled at the end of each stage of the experiment (~70 and ~110 kg). Blood was drawn from 54 barrows (9 from each group—one from each replicate). The animals had no access to feed for 12 h before blood was drawn. Blood was collected from the jugular vein into 10 mL heparinized test tubes.

After final weighing (~70 and ~110 kg) at slaughter, n = 9 barrows from each experimental group were randomly selected (one barrow per pen with body weight closest to the average body weight of the group) for analysis of mineral contents in the bone and in chyme from the intestine. Only barrows were used in order to avoid any sex-dependent differences in bone traits, as sex hormones may affect bone mineralization [16,17,18,19,20,21,22]. The right femur was removed, cleaned of adherent tissues, wrapped in gauze soaked in 0.9% saline, and frozen at −20 °C until analysis.

For balance and digestibility tests during the growing (65–70 kg BW) and finishing (105–110 kg BW) periods, 9 barrows from each group were placed in metabolic cages (a separate cage for each group) for collection of urine and feces. Urine flowed into special plastic containers placed under the cages. Every day, 10 mL of 10% sulfuric acid was poured into the containers to bind ammonia. Feces were retained on plastic mesh placed under the slats in the pens. For four days, feces and urine were collected at the same time of day and weighed. From the daily collection of feces and urine, about 20% was placed in special jars with ground glass stoppers (urine) and plastic bags (feces). The samples were refrigerated at 4 °C. The urine and feces collected over 4 days were mixed thoroughly, and 1 kg samples of feces and 1 L samples of urine were collected. The averaged samples prepared in this manner were used for chemical analyses.

Dry matter content was determined in the samples of feces and chyme, and the contents of P, Ca, Mg, Cu, Zn, and Fe were determined in samples of plasma, dried feces, and urine by using standard AOAC [14] methods.

### 2.5. Statistical Analysis

Statistical analysis of the data was performed by using Statistica software, version 13.3.721.0 (StatSoft Poland Sp. z o.o., Kraków, Poland, 2022, www.statsoft.pl) (accessed on 10 March 2022). A pig was the experimental unit for all analyses. Normality of data and homogeneity of variances were tested by using the Shapiro–Wilk and Levene’s tests, respectively. Data were analyzed by one-way ANOVA. Means were separated by using Tukey’s HSD test. Orthogonal polynomial contrasts were performed between the phosphorus-deficient treatments (NC, NC + 250 FTU/kg, NC + 500 FTU/kg, NC + 1000 FTU/kg, and NC + 1500 FTU/kg) to test linear and quadratic responses to the inclusion of increasing levels of phytase to the diets. Pearson’s correlation test was performed to determine the linear correlations between the contents of minerals in the small intestinal chyme, blood, femur, feces, and urine. The following scale was used to interpret Pearson’s r correlation coefficients: 0 < r < 0.3—low correlation; 0.3 ≤ r < 0.5—moderate correlation; 0.5 ≤ r < 1—high correlation (Supplementary Tables S1–S3). In all analyses, a *p*-value of less than 0.05 was considered statistically significant. All data are expressed as means and SEM (standard error of the means).

## 3. Results

### 3.1. Influence of Phytase Level on the Content of Minerals in Chyme from the Small Intestine of Pigs

The content of minerals in the small intestinal chyme is presented in Table 2. All groups of pigs receiving the phosphorus-deficient NC diet and the phosphorus-deficient diet with added phytase had similar concentrations of phosphorus and calcium in the small intestinal chyme (*p* > 0.05). In the case of phosphorus content in the intestine of pigs during the growing period, there was a linear decrease as the level of phytase increased (*p* = 0.003); however, this effect was not observed during the second fattening period (*p* > 0.05). The highest contents of both phosphorus (*p* < 0.001) and calcium (*p* < 0.05) were noted in the intestine of pigs in the PC group in both fattening periods. The content of magnesium in the intestinal chyme was also significantly higher in the PC pigs than in the other experimental groups throughout the fattening period (*p* < 0.05). Magnesium content decreased linearly in the pigs fed a phosphorus-deficient diet with added phytase (*p* < 0.001), reaching the lowest value in pigs fed a diet with 1000 and 1500 FTU/kg, as confirmed by the value of the quadratic effect (*p* < 0.001). 

In the case of the other analyzed minerals (Cu, Zn, and Fe) in the chyme of the small intestine, no significant differences were noted between treatments (*p* > 0.05).

### 3.2. Effect of Phytase Level on the Content of Minerals in the Blood Plasma of Pigs

The effect of increasing the amount of phytase in the P-deficient diets on the level of minerals in the blood plasma is presented in Table 2. Pigs fed the PC and phosphorus-poor diets with added phytase, irrespective of the amount added, had significantly higher plasma levels of phosphorus and calcium than group NC in both fattening periods (in growing period, *p* < 0.001 for treatment, phytase, and phytase contrasts effects for both phosphorus and calcium; in finishing period, *p* < 0.001 for treatment, phytase, and linear contrast effects and *p* < 0.05 for quadratic contrast for phosphorus; *p* < 0.01 and *p* < 0.05 for treatment and phytase effect, respectively, for calcium). The highest levels of phytase supplementation (1000 and 1500 FTU/kg) caused a significant increase in the plasma levels of Cu and Zn, in comparison to group NC (by about 16% and 50%, respectively), in both fattening periods (linear, *p* < 0.01). Cu and Zn concentrations in groups NC and PC were similar throughout the fattening period. All groups of pigs had similar plasma levels of magnesium and iron in both the growing and finishing periods (*p* > 0.05). However, their content increased linearly in pigs fed a phosphorus-deficient diet with added phytase (*p* < 0.05).

The content of minerals in the femur is presented in Table 3. As the level of added phytase increased in finishing pigs fed a phosphorus-deficient diet, a linear and a quadratic (*p* < 0.05) increase was observed in the content of phosphorus in the femur, while only a quadratic increase was noted during the growing period (*p* < 0.01). In both the growing and finishing periods, pigs fed the PC diet and the phosphorus-deficient diet supplemented with 500 FTU/kg phytase had significantly higher P levels in the femur as compared to group NC and the groups receiving a phosphorus-deficient diet supplemented with 250 and 1000 FTU/kg phytase (*p* < 0.05). In both fattening periods, the content of calcium and magnesium in the femur was similar in all experimental groups.

### 3.3. Effect of Phytase Level on Femur Mineral Concentrations

The concentration of copper in the femur of pigs during the growing period was significantly higher in groups NC + 500 and NC + 1000 than in the remaining experimental groups (*p* < 0.001). Both a linear (*p* = 0.002) and a quadratic (*p* < 0.001) effect were observed for the increase in Cu. The content of copper in the femur was significantly higher in finishing pigs from the groups fed a phosphorus-deficient diet supplemented with 250 and 500 FTU/kg, and also in the pigs from group PC, as compared to group NC and the groups receiving feed supplemented with 1000 and 1500 FTU/kg (*p* < 0.001). The content of zinc in the femur of pigs in the growing period was significantly higher in groups NC + 500 and NC + 1000 than in the remaining experimental groups (*p* < 0.001). The data showed a linear (*p* < 0.001) and a quadratic (*p* < 0.001) effect. The femurs of pigs in the treatment group with NC + 1000 had a significantly higher content of Zn than those of pigs fed the NC finisher diet (by 16.7%), but the Zn content increased with the phytase level, showing both a linear and a quadratic response (*p* = 0.050 and *p* = 0.029, respectively). The content of iron in the femur was significantly lower in pigs fed the phosphorus-deficient control diet and the diet supplemented with 1000 and 1500 FTU/kg compared to the other experimental groups (*p* < 0.01), showing both a linear and a quadratic response (*p* < 0.05) in both fattening periods.

### 3.4. Effect of Phytase Level on Urinary and Fecal Mineral Excretion

The content of minerals in the feces of growing–finishing pigs is presented in Table 4. The content of phosphorus and calcium in the feces was significantly higher in pigs fed the PC diet in comparison to the remaining experimental groups in both fattening periods (*p* < 0.001). There was a linear decrease in the content of phosphorus (*p* < 0.001) and calcium (*p* < 0.05) in the feces of pigs fed a phosphorus-deficient diet with increasing levels of phytase during the growing period. During the finishing period, there was a linear decrease in the phosphorus content (*p* < 0.001), as well as a quadratic response (*p* = 0.003). A quadratic response was also noted in the case of calcium content in the feces of pigs in the finishing period (*p* < 0.001). The content of magnesium in the feces of pigs was significantly higher in the PC group than in the other experimental groups (*p* < 0.001). There was a linear decrease in magnesium content in the pigs fed a phosphorus-deficient diet with added phytase (*p* = 0.002), and for pigs fed a diet with 1500 FTU/kg, its value was lower than that noted in groups PC and NC (*p* < 0.001). The magnesium content in the feces during the finishing period showed a quadratic response (*p* < 0.01). Although the ANOVA *p*-value did not show an effect of the dietary treatment on the content of copper in the feces of pigs during the growing period (*p* > 0.05), the contrast analysis showed a linear decrease in Cu content as the phytase level increased in the phosphorus-deficient diets (*p* = 0.049). An increase in the level of phytase caused a linear decrease in the content of zinc in the feces of pigs in both fattening periods (*p* < 0.001), with significantly higher zinc content noted in the feces of growing–finishing pigs in groups NC and PC. No significant differences were noted in iron content in the feces between groups in the entire fattening period.

The content of minerals in the urine of growing–finishing pigs is presented in Table 4. Among all dietary treatments, the highest phosphorus content in the urine was observed in growing–finishing pigs fed the PC diet (*p* < 0.001). At the same time, a linear decrease in phosphorus content was observed in the urine of pigs fed a phosphorus-deficient diet supplemented with increasing levels of phytase (*p* < 0.001). A linear decrease in calcium content in the urine of growing–finishing pigs was observed as the level of phytase increased (*p* < 0.001), with the highest Ca content noted in the urine of pigs fed the PC, NC, and NC + 250 diets (*p* < 0.001). Pigs fed phosphorus-deficient diets supplemented with 1000 and 1500 FTU/kg had a significantly higher content of magnesium in the urine during the finishing period compared to the other experimental groups (*p* < 0.001). In the case of the phosphorus-deficient diets, the effect of the increase in magnesium content in the urine was linear (*p* < 0.001). Increasing the level of phytase decreased the copper content in the urine of growing–finishing pigs (linear and quadratic, *p* < 0.001 for both effects). Significantly, the highest copper content in the urine was noted in the groups NC and PC. A quadratic and linear decrease in the content of zinc was noted in the urine of growing–finishing pigs fed a phosphorus-deficient diet supplemented with phytase (*p* < 0.05). The lowest zinc content in the urine of growing pigs was noted in pigs fed the NC and PC diets, but this was not observed in the finishing pigs. The iron content in the urine was significantly influenced by the dietary treatment (*p* < 0.001). In the case of phosphorus-deficient diets, this effect was linear (*p* < 0.01) in both fattening periods and quadratic in the first period (*p* < 0.001). The lowest iron content was noted in the urine of growing–finishing pigs receiving a phosphorus-deficient diet with 1500 FTU/kg.

### 3.5. Correlations between the Content of Minerals in the Small Intestinal Chyme, Blood, Femur, Feces, and Urine

**Figure 1:**
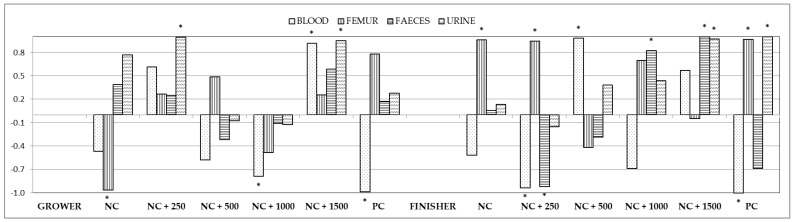
Correlations between content of phosphorus in the chyme and in the blood, femur, feces, and urine. Asterisks indicate significant correlations at *p* < 0.05.

Correlation coefficients between phosphorus content in the chyme of the small intestine and in the blood, femur, feces, and urine were calculated, as well (Figure 1; Supplementary Table S1). Pearson’s correlation coefficient (r) between the level of phosphorus in the chyme and its content in the blood confirmed a significantly (*p* < 0.05) strong negative correlation in group PC during the whole fattening period, as well as for group NC + 1000 during the growing period and group NC + 250 in the finishing period. A high, but, in this case, positive correlation (*p* < 0.05) was noted for groups NC + 1500 (growing period) and NC + 500 (finishing). A high negative correlation was observed between the phosphorus content in the chyme and in the femur in group NC during the growing period. The correlation was equally strong in the finishing period, but positive (*p* < 0.05). A positive and strong correlation was also observed for these variables in the finishing period in groups NC + 250 and PC. Significant strong correlations between phosphorus content in the chyme and in the feces were observed only in the second fattening period for groups NC + 250 (negative) and for groups NC + 1000 and NC + 1500 (positive). Strong positive correlations were also noted between phosphorus content in the chyme of the intestine and in the urine in groups NC + 250 and NC + 1500 (first fattening period) and in groups NC + 1500 and PC (second fattening period).

**Figure 2:**
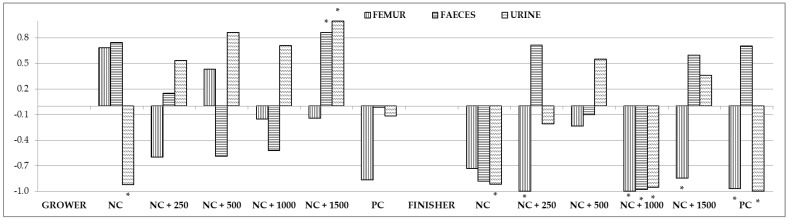
Correlations between content of phosphorus in the blood and in the femur, feces, and urine. Asterisks indicate significant correlations at *p* < 0.05.

The correlation coefficients estimated for phosphorus content in the blood and in the femur, feces, and urine are presented in Figure 2 and Supplementary Table S2. No significant correlations were noted between the phosphorus content in the blood and its content in the femur. However, strong correlations were observed between its content in the blood and in the feces in group NC + 1500 (positive correlation) in the growing period and in group NC + 1000 (negative correlation) in the finishing period. Phosphorus concentrations in the blood and urine were also strongly correlated in groups NC (negative correlation) and NC + 1500 (positive correlation) in the growing period and in groups NC, NC + 1000, and PC (negative correlation) in the final period of fattening.

**Figure 3:**
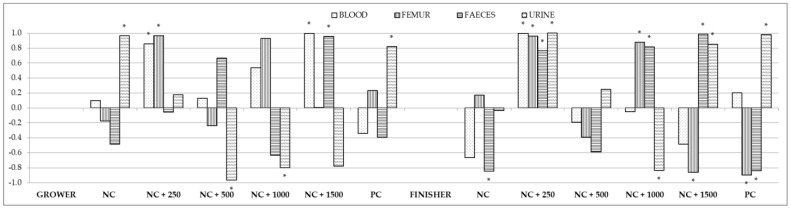
Correlations between content of calcium in the chyme and in the blood, femur, feces, and urine. Asterisks indicate significant correlations at *p* < 0.05.

Correlation coefficients were also calculated for the calcium content in the chyme and in the blood, femur, feces, and urine (Figure 3; Supplementary Table S1). A strong positive correlation between the calcium content in the chyme and in the blood was noted in group NC + 250 in the entire fattening period. In group NC + 1500, such a correlation was noted only in the first fattening period. The calcium content in the chyme was strongly positively correlated with its content in the femur in group NC + 250 in the entire fattening period. A similar correlation was observed in group NC + 1000, but only in the second fattening period. Strong negative correlations were noted between these variables in the second fattening period for groups NC + 1500 and PC. Strong positive correlations between the level of calcium in the chyme and in the feces were noted for group NC + 1500 in the entire fattening period. In the second period of fattening, a strong positive correlation was also observed between these variables in group NC + 1000, and a strong negative correlation in groups NC and PC. The calcium content in the chyme and in the urine was strongly positively correlated in group PC and strongly negatively correlated in group NC + 1000 throughout the fattening period. A strong correlation for these variables was also observed in groups NC (positive correlation) and NC + 500 (negative correlation) in the first period of fattening and in groups NC + 250 and NC + 1500 (positive correlation) in the second period.

**Figure 4:**
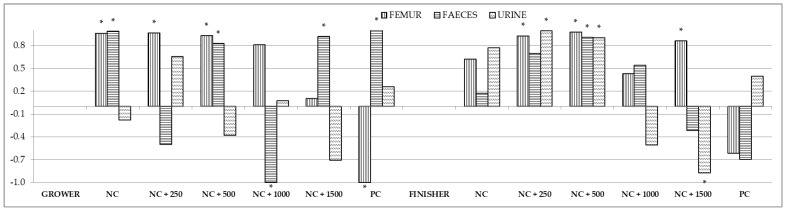
Correlations between content of calcium in the blood and in the femur, feces, and urine. Asterisks indicate significant correlations at *p* < 0.05.

The coefficients of correlation between the content of calcium in the blood and in the femur, feces, and urine are presented in Figure 4 and Supplementary Table S2. A strong positive correlation was observed for the content of calcium in the blood and in the femur in groups NC + 250 and NC + 500 throughout the fattening period, as well as for NC only in the first fattening period and for NC + 1500 only in the second period. In contrast, in group PC a strong negative correlation was noted for these variables. The calcium contents in both the blood and feces were also positively correlated in group NC + 500 throughout the fattening period and in groups NC, NC + 1500, and PC only in the first period. A strong correlation was noted in group NC + 1000, but it was a negative one, and it was detected only in the first fattening period. Only in the second period were correlations noted between calcium content in the blood and urine in groups NC + 250 and NC + 500 (positive correlations) and in group NC + 1500 (negative correlation).

## 4. Discussion

Studies show that only about 20–30% of P and 35–60% of Ca are available from compound feeds for pigs, but their availability increases significantly following the use of phytase. In this experiment, we analyzed the effect of phytase in a range of 0–1500 FTU/kg of diet without an inorganic source of phosphorus in order to assess the effect on levels of P, Ca, Mg, Cu, Zn, and Fe. The analyzed amounts of phytase used in the diet of pigs in all groups were close to the values planned in the experiment.

The analyzed concentrations of minerals in the positive control diet (PC) were higher than in the negative control diet (NC) or in the diets with phytase. This was expected, due to the lack of dicalcium phosphate in these diets, as commercial sources of dicalcium phosphate, apart from Ca and P, also contain a number of other micro-minerals [23].

The study showed that, the higher the phytase activity in the diet, the lower the concentration of phosphorus, calcium, and magnesium in the chyme. This is supported by research by Madrid et al. [24], in which supplementation of a low-P diet with microbial phytase (500 FTU/kg) proved to be an effective method of increasing the availability not only of P, but also of Ca, Mg, and Cu. Several studies have demonstrated that phytase supplementation of diets with low P content increases the digestibility of P and Ca [25]. Research by Arredondo et al. [23] has also shown that, as phytase activity increases in a maize and soybean diet without inorganic P, the availability of most macro- and microelements increases.

According to Lei et al. [26], the plasma concentration of P is a sensitive and accurate measure of the effect of phytase on utilization of phytate P. As retention of P and Ca improved as a result of phytase supplementation, the plasma concentrations of P and Ca were expected to increase. The increase in the content of these elements is due to the increased hydrolysis of phytate complexes, which release them during passage through the intestines. In our experiment, the increase in plasma content of P relative to group NC was most pronounced following the use of 500 FTU/kg, while an increase in the concentration of Ca in the plasma of both growing and finishing pigs was noted in all groups receiving phytase. Gentile et al. [27] and Jendza et al. [28] observed a similar pattern in weaned piglets fed a diet poor in P and supplemented with *A. niger* phytase. The increase in the P concentration in the plasma is a consequence of its increased bioavailability (digestibility and retention). This is confirmed by the significant negative correlation between P content in the chyme and in the plasma following application of phytase in the amount of 1000 FTU/kg (NC + 1000) during the growing period and 250 FTU/kg during the finishing period. Hong and Kim [10], however, reported a non-linear dependence of phosphorus digestibility on the level of phytase in the diet. They suggested that the limited number of substrates in diets may contribute to the non-linear nature of the relationship. Similar observations have been made by Rosenfelder-Kuon et al. [29] and by Sung and Kim [30].

Calcium absorption, which takes place in the small intestine, is only slightly dependent on the concentration of Ca and P in the diet. However, an excess or deficiency of one of the minerals impairs the utilization of the other [31]. During absorption, Ca is stored in the intestinal wall and competes with Mg, which is not stored. When absorbed Ca exceeds demand, the excess is excreted in the urine [32,33]. This was probably observed in our experiment in group PC, with the addition of inorganic sources of Ca, where a high positive correlation was noted between Ca content in the chyme and in the urine throughout the fattening period. In the case of supplementation with phytase in the amount of 1000 FTU/kg, the corresponding correlation was negative: throughout the fattening period, there was a high negative correlation between the Ca content in the chyme and in the urine. Tsai et al. [2] reported a similar effect when using a P-deficient diet supplemented with phytase in barrows. The addition of phytase completely eliminated the loss of Ca in the urine. The increase in P and Ca in the plasma of pigs from the group receiving a diet with *Aspergillus oryzae* may indicate an optimal ratio of Ca to non-phytin phosphorus in the diets. Increasing the level of Ca in the diet of pigs and adding phytase [34] increases the absorption of Ca. The apparent total tract digestibility of Ca is not affected by an increase in the Ca level relative to the requirement, but it is affected by a decrease in the Ca level, because the endogenous loss of Ca represents a greater proportion of the Ca output [33,35]. The level of magnesium may also directly decrease absorption of Ca, as both elements use a common transport mechanism, which explains the low bioavailability of Ca in dolomitic limestone [31]. The interactions of calcium and phytase have been analyzed in detail by Selle and Ravindran [36].

The linear decrease in the amount of magnesium in the chyme of the growing and finishing pigs receiving a diet with phytase produced by a genetically modified strain of *Aspergillus oryzae* corresponded to its level in the plasma (linear relationship). The increase in the absorption and retention of this element when phytase activity in the diet increased is in agreement with research by Zeng et al. [37].

Phytase is also believed to play a role in increasing the bioavailability of other divalent elements released from phytin complexes, i.e., Cu, Zn, Fe, and others [35,38].

No changes were noted in the concentrations of Cu, Zn, or Fe in the chyme relative to group PC. However, the content of Cu and Zn in the plasma of both growing and finishing pigs increased linearly. This relationship was particularly evident in the case of higher levels of phytase in the diets, i.e., 1000 and 1500 FTU. This can be attributed to the significant negative correlation between the content of Cu and Zn in the chyme and in the blood at phytase levels of 1000 FTU for zinc, 1500 FTU for Cu (growing period), and 500 FTU for both elements (finishing period). An increase in Cu absorption as the level of phytase in the diets increased was documented in some earlier studies [39]. The presence of phytase produced by a genetically modified strain of *Aspergillus oryzae* also caused significant changes in the plasma level of iron in the pigs. The effect of phytase on Fe absorption corresponds with some earlier data [23,40]. However, the effect of phytase was smaller than that observed in the case of Cu. This may have been linked to the higher resorption of Cu from the intestinal lumen. Phytate binds divalent cations in order of decreasing stability. Fe^2+^ is the least likely to bind to phytate, and this may explain the less pronounced response to the addition of phytase in the case of Fe absorption in this experiment. In addition, Fe and Cu have antagonistic effects due to competition for absorption sites in the intestinal mucosa [41]. Interference of Cu in the absorption of Fe involves the formation of complexes [42]. Other factors also take part in the absorption of iron, such as acidification of the chyme and the presence of the specific protein apoferritin, which takes part in further transformations of iron and its transfer to the plasma globulin transferrin. Therefore, further studies should take these factors into account when analyzing the effect of phytase on intestinal absorption of iron. Zinc is also an antagonistic element in relation to iron. Therefore, disruption of Fe absorption in the small intestine may also have been limited by increased absorption of zinc. Transport mechanisms are generally highly specific for a given mineral and easily become saturated, meaning that they can transport only a limited amount of mineral in a given period of time [43]. In some cases, the efficiency of these transport systems can be increased when the body needs a specific mineral or decreased when its reserves are sufficient.

Minerals absorbed into the blood are used in biochemical processes, including the formation of bone tissue. According to O’Doherty et al. [44], the content of Ca and P in the bones is a very good indicator of their status. Deposition of Ca in bone tissue depends on its level in the diet [31]. In this experiment, however, varied supplementation with phytase did not improve the content of Ca in the bone in comparison with the groups without phytase. This may have been linked to plasma content of calcium that was in agreement with reference values [45], as indicated by the statistically significant positive correlation between calcium content in the blood and in the femur in the groups receiving 250, 500, and 1500 FTU). According to González-Vega and Stein [31], fluctuations in the content of Ca in the plasma result in increased (high Ca level in the blood) or decreased (low Ca level in the blood) release of Ca from the bones [31]. Many studies indicate an increase in Ca content in the bones due to the use of phytase in the diet [38,46]. 

The use of phytase increased the content of phosphorus and magnesium in the femur, i.e., elements with an active role in metabolic processes of bone tissue associated with mineralization and collagen synthesis. About 85–90% of total P accumulated in the bones comes from absorption in the small intestine, where there is a linear proportional relationship between its absorption and intake in the diet, in contrast to Ca absorption [47]. The total amount of P increased with the level of phytase in the diets, thus showing that the inclusion of phytase in the diet makes more P available for absorption. This observation is in agreement with data reported by Petersen et al. [46]. The improvement in the absorption of phosphorus in the femur of finishing pigs receiving a phytase supplement is also indicated by the negative correlation between phosphorus content in the blood and the femur, especially when phytase was used in the amounts of 1000 and 1500 FTU, but also at 250 FTU. In addition, the phytase diet, while improving absorption of phosphorus, increased its excretion in the feces, as confirmed by numerous reports [27].

An intake of P above the daily standard can increase losses of bone mass. If this is accompanied by a Ca deficiency, P activates a mechanism of Ca release from the bones, regulating calcium balance. It should be emphasized that excessive P intake in the daily diet inhibits Ca absorption from the gastrointestinal tract, and the excess P is excreted in the urine [47,48,49].

The presence of phytase, by improving the bioavailability of Zn, caused an increase in the content of Zn not only in the plasma, but also in the femur. This was particularly evident in the case of phytase in the amount of 1000 FTU. Similar observations were reported by Revy et al. [50].

According to Tsai et al. [2], nearly 95.5% of phosphorus excreted by pigs whose feed does not contain phytase is lost in the feces, with minimal loss in urine (4.5%). Microbial phytase is a factor that reduces excretion of phosphorus into the environment. In commercial diets, the use of a phytase supplement in the amount of 500–700 FTU/kg of diet is recommended [1]. Many studies have shown that phytase used in amounts of up to even 2000 FTU/kg reduces excretion of phosphorus and calcium [51,52].

In our experiment, a significant decrease in the amount of phosphorus excreted in the feces and also in the urine was obtained following the addition of phytase from a genetically modified strain of *Aspergillus oryzae* in the amounts of 1000 and 1500 FTU to diets low in phosphorus. This was noted in both the growing and finishing periods, and the relationship was linear. The linear decrease in excretion of phosphorus and the increase in absorption and retention of this element due to the presence of phytase in the diet were consistent with research conducted by Jendza and Adeola [53] and Tsai et al. [2]. Microbial phytase also affects utilization of calcium [54]. Calcium is absorbed independently of the content of Ca and P in the diet, but if Ca is absorbed in amounts exceeding demand, the excess is excreted in the urine [32,33].

The linear decrease in calcium excretion and its increased absorption and retention in the presence of phytase from a genetically modified strain of *Aspergillus oryzae* were in agreement with research by She et al. [38]. According to Stein et al. [32], pigs fed a low-phosphate diet were able to increase intestinal absorption of Ca and decrease its amount in the feces and urine. The amount of Ca that reaches the kidneys and is recirculated or excreted in the urine is influenced by the levels of Ca and P in the diet [32,33]. The use of phytase produced by a genetically modified strain of *Aspergillus oryzae* also caused a linear decrease in the amount of Mg excreted in the feces. The role of microbial phytase in reducing excretion of Mg in the feces is described by Woyengo et al. [55]. Similar results were obtained by Zeng et al. [37].

The activity of microbial phytase also involves the release of divalent elements such as Zn or Cu from phytin complexes and an increase in their bioavailability, thus reducing their excretion into the environment [5]. It should be noted that Cu and Zn are two trace minerals that are regulated in Europe in order to reduce emissions from pig waste and, thereby, reduce environmental pollution (regulation (WE) no. 166/2006 of the European Parliament and of the Council). The phytase used in this study, produced by a genetically modified strain of *Aspergillus oryzae*, significantly decreased mainly zinc content in the feces throughout the experiment. The linear decrease in excretion of zinc in the feces was significant in the case of high levels of phytase, i.e., 1000 and 1500 FTU. Copper and iron behaved somewhat differently; their levels in pigs fed diets supplemented with phytase were lower only in the urine. At the same time, however, the Cu contents in the plasma and the feces were strongly negatively correlated in the case of phytase supplementation at 1000 FTU throughout the fattening period. Cu is primarily excreted in the feces, and only to a minor extent in the urine [56]. Bikker et al. [40], in a meta-analysis of published experiments, found no effect of the inclusion of 3-phytase from *Aspergillus niger* on the digestibility and plasma level of Cu. This may indicate that the 6-phytase used in our study, produced by a genetically modified strain of *Aspergillus oryzae*, is more effective.

This experiment has certain limitations that should be taken into account in further studies. Specifically, subsequent research should include analyses of hormones, vitamins (D_3_), and transporters taking part in mineral homeostasis or the utilization of individual elements in the body. On the other hand, in our opinion, this study has many strengths: (1) several concentrations of phytase; (2) two periods, namely grower and finisher; (3) both basic macroelements and several microelements important for systemic homeostasis; and (4) various types of biological material in which minerals’ concentrations were analyzed.

## 5. Conclusions

Proper functioning of the skeletal system depends on the intake of calcium and other minerals (such as magnesium, zinc, and phosphorus), which cause bone mineralization. This can be achieved through the use of phytase in the diet of growing pigs.

Our previous research [9] showed that including phytase in the amount of 500 FTU/kg or higher improved the feed conversion ratio, while, at 1000 FTU/kg, it increases daily weight gains in fatteners. At the same time, 500 FTU/kg was sufficient to improve bone parameters (increased P content in the metacarpal bone and the mechanical strength of the femur).

Expanding the research to encompass additional aspects of the effect of phytase on the mineral status of fattening pigs and the excretion of elements into the environment (P, Ca, Mg, Cu, Zn, and Fe) made it possible to determine the optimal level of phytase inclusion in the diet. Our study on the use of phytase in a range of 0–1500 FTU/kg in low-phosphorus diets for fattening pigs indicates that 1000 FTU is the most effective level of phytase in terms of increasing absorption from the gastrointestinal tract and deposition by the bones, as well as reducing excretion of elements into the environment. 

## Figures and Tables

**Table 1:** Dry matter and crude ash content, mineral concentrations, and total phytase activity (FTU/kg) in grower and finisher pig diets [9].

Treatment ^1^	NC	NC + 250	NC + 500	NC + 1000	NC + 1500	PC
Grower						
Dry matter (g/kg)	876.5	873.0	879.8	880.3	882.3	87.93
Crude ash (g/kg)	41.1	41.2	41.5	39.8	40.2	41.1
Calcium (g/kg)	5.51	5.45	5.47	5.49	5.57	6.62
Total phosphorus (g/kg)	4.82	4.74	4.75	4.78	4.83	5.69
Magnesium (g/kg)	1.82	1.83	1.82	1.83	1.84	1.82
Iron (mg/kg)	120.4	120.3	120.4	120.5	120.3	120.4
Zinc (mg/kg)	135.8	135.5	135.9	135.4	135.8	135.7
Copper (mg/kg)	20.3	20.1	20.2	20.3	20.2	20.3
Phytase activity (FTU/kg)	187	411	639	1081	1574	195
Finisher						
Dry matter (g/kg)	871.6	867.7	869.2	871.3	868.9	869.7
Crude ash (g/kg)	42.6	43.2	42.8	43.9	40.1	43.6
Calcium (g/kg)	4.94	4.92	4.98	4.95	4.94	6.09
Total phosphorus (g/kg)	4.25	4.23	4.29	4.27	4.31	5.27
Magnesium (g/kg)	1.76	1.77	1.76	1.78	1.77	1.76
Iron (mg/kg)	105.4	105.7	105.3	105.8	105.7	105.4
Zinc (mg/kg)	124.3	125.1	124,6	125.2	124.6	124.7
Copper (mg/kg)	19.4	18.9	18.8	19.2	19.3	19.4
Phytase activity (FTU/kg)	209	394	699	1160	1577	205

^1^ Diets 1–5 were phosphorus-deficient, with 0 (NC), 250, 500, 1000, or 1500 phytase units FTU/kg, respectively. A sixth diet (positive control—PC) was formulated with increased dicalcium phosphate to meet the nutrient requirements of the pigs [13].

**Table 2:** Mineral content in the ileum and blood plasma of growing–finishing pigs.

Treatment ^1^	P	Ca	Mg	Cu	Zn	Fe	P	Ca	Mg	Cu	Zn	Fe
	Grower						Finisher					
Ileum	g/kg DM	g/kg DM	g/kg DM	mg/kg DM	mg/kg DM	mg/kg DM	g/kg DM	g/kg DM	g/kg DM	mg/kg DM	mg/kg DM	mg/kg DM
NC ^2^	9.60 ^b^	11.03 ^b^	2.69 ^b^	55.94	496.2	564.6	10.09 ^b^	11.97 ^b^	2.93 ^b^	56.91	536.8	573.6
250 ^3^	9.30 ^b^	10.95 ^b^	2.63 ^b^	55.52	491.3	563.4	9.90 ^b^	11.77 ^b^	2.92 ^b^	55.28	531.2	572.8
500 ^3^	9.13 ^b^	10.84 ^b^	2.38 ^c^	55.39	497.7	565.9	9.94 ^b^	11.54 ^b^	2.91 ^b^	51.78	532.1	580.6
1000 ^3^	8.98 ^bc^	10.74 ^b^	2.47 ^c^	55.41	499.4	555.4	9.77 ^b^	11.45 ^b^	2.84 ^bc^	52.11	526.7	573.4
1500 ^3^	8.92 ^c^	10.71 ^b^	2.16 ^d^	55.12	495.2	553.7	9.80 ^b^	11.61 ^b^	2.87 ^bc^	51.73	525.2	578.3
PC ^4^	11.11 ^a^	12.56 ^a^	2.99 ^a^	55.97	497.4	571.9	12.04 ^a^	13.07 ^a^	3.03 ^a^	56.85	538.4	581.1
SEM ^5^	0.089	0.320	0.041	0.981	7.70	10.94	0.396	0.486	0.018	3.63	8.46	6.50
*p*-value												
TRT ^6^	<0.001	0.003	<0.001	0.987	0.988	0.610	<0.001	0.027	0.026	0.547	0.992	0.998
PHY ^7^	0.051	0.642	0.001	0.982	0.967	0.672	0.954	0.815	0.009	0.509	0.991	0.996
Linear ^8^	0.003	0.125	<0.001	0.557	0.840	0.207	0.471	0.328	<0.001	0.109	0.626	0.766
Quadratic ^8^	0.429	0.850	<0.001	0.921	0.938	0.613	0.862	0.488	<0.001	0.520	0.976	0.882
Plasma	mmol/L	mmol/L	mmol/L	µmol/L	µmol/L	µmol/L	mmol/L	mmol/L	mmol/L	µmol/L	µmol/L	µmol/L
NC ^2^	1.63 ^c^	1.79 ^b^	0.694	30.27 ^b^	10.09 ^b^	40.10	1.93 ^c^	1.70 ^b^	0.741	31.40 ^b^	10.79 ^b^	40.45
250 ^3^	2.39 ^ab^	2.17 ^a^	0.746	30.57 ^b^	11.38 ^b^	39.90	2.05 ^b^	2.18 ^a^	0.748	31.02 ^b^	11.11 ^b^	39.92
500 ^3^	2.49 ^a^	2.16 ^a^	0.722	32.38 ^ab^	11.92 ^b^	41.17	2.14 ^ab^	2.05 ^a^	0.743	33.97 ^ab^	12.19 ^b^	41.93
1000 ^3^	2.29 ^ab^	2.17 ^a^	0.744	35.93 ^a^	16.19 ^a^	41.28	2.10 ^ab^	2.12 ^a^	0.753	36.49 ^a^	14.37 ^a^	41.02
1500 ^3^	2.13 ^b^	2.14 ^a^	0.723	34.27 ^a^	14.21 ^a^	41.88	2.15 ^ab^	2.14 ^a^	0.741	35.48 ^a^	14.56 ^a^	42.62
PC ^4^	2.33 ^ab^	2.19 ^a^	0.743	33.59 ^ab^	11.51 ^b^	39.45	2.21 ^a^	2.17 ^a^	0.750	31.63 ^b^	11.38 ^b^	40.12
SEM ^5^	0.050	0.025	0.009	0.687	0.533	0.305	0.018	0.017	0.005	0.506	0.342	0.320
*p*-value												
TRT ^6^	<0.001	<0.001	0.591	0.011	0.002	0.071	<0.001	0.009	0.973	<0.001	<0.001	0.072
PHY ^7^	<0.001	<0.001	0.310	0.055	0.004	0.094	<0.001	0.020	0.940	0.002	<0.001	0.096
Linear ^8^	<0.001	<0.001	<0.001	0.009	0.001	0.039	<0.001	0.097	<0.001	<0.001	<0.001	0.028
Quadratic ^8^	<0.001	<0.001	0.173	0.684	0.453	0.554	0.036	0.458	0.619	0.542	0.538	0.621

^a–c^ Means with the same superscript letters are statistically the same across all 6 treatments (*p* < 0.05) based on Tukey’s post hoc test. ^1^ Diets 1–5 were P-deficient diets with 0 (NC), 250, 500, 1000, or 1500 phytase units FTU/kg, respectively. A sixth diet (positive control; PC) was formulated with increased dicalcium phosphate to meet the nutrient requirements of the pigs. ^2^ NC—negative control. ^3^ Phytase level added to NC. ^4^ PC—positive control. ^5^ SEM—standard error of the means. ^6^ The *p*-value for overall effect of dietary treatment (diets 1–6). ^7^ The *p*-value for phytase effect in P-deficient diets (diets 1–5). ^8^ Orthogonal polynomial (linear and quadratic) contrasts were performed to test the effect of phytase level in the P-deficient diets (diets 1–5).

**Table 3:** Mineral content in dry matter of femurs from growing (70 kg BW) and finishing (110 kg BW) pigs.

Treatment ^1^	P g/kg	Ca g/kg	Mg g/kg	Cu mg/kg	Zn mg/kg	Fe mg/kg	P g/kg	Ca g/kg	Mg g/kg	Cu mg/kg	Zn mg/kg	Fe mg/kg
Grower							Finisher					
NC ^2^	196.6 ^b^	329.0	4.18	0.397 ^b^	167.6 ^c^	11.66 ^a^	213.7 ^b^	356.5	4.54	0.394 ^b^	185.3 ^b^	10.77 ^a^
250 ^3^	197.8 ^b^	330.6	4.19	0.446 ^b^	170.9 ^bc^	12.04 ^a^	216.9 ^b^	359.0	4.56	0.591 ^a^	198.0 ^ab^	11.91 ^a^
500 ^3^	204.7 ^a^	333.1	4.17	0.508 ^a^	177.2 ^a^	12.65 ^a^	245.2 ^a^	363.0	4.53	0.688 ^a^	207.8 ^ab^	12.85 ^a^
1000 ^3^	198.3 ^b^	330.0	4.20	0.470 ^a^	180.8 ^a^	10.47 ^b^	222.7 ^b^	356.0	4.71	0.425 ^b^	222.5 ^a^	7.14 ^b^
1500 ^3^	198.9 ^ab^	331.8	4.19	0.462 ^ab^	175.5 ^ab^	10.21 ^b^	230.1 ^ab^	367.5	4.63	0.352 ^b^	198.3 ^ab^	8.21 ^b^
PC ^4^	204.3 ^a^	333.6	4.18	0.399 ^b^	169.1 ^c^	11.76 ^a^	239.6 ^a^	349.5	4.72	0.581 ^a^	189.0 ^ab^	11.83 ^a^
SEM ^5^	0.635	0.807	0.014	0.008	0.806	0.131	4.34	3.93	0.153	0.051	7.71	0.747
*p*-value												
TRT ^6^	<0.001	0.546	0.994	<0.001	<0.001	<0.001	0.001	0.146	0.833	<0.001	0.033	0.001
PHY ^7^	<0.001	0.655	0.980	<0.001	<0.001	<0.001	0.004	0.394	0.842	<0.001	0.040	0.002
Linear ^8^	0.178	0.447	0.764	0.002	<0.001	<0.001	0.030	0.213	0.427	0.139	0.050	0.004
Quadratic ^8^	0.003	0.508	0.976	<0.001	<0.001	<0.001	0.041	0.691	0.980	<0.001	0.029	0.044

Note: See Table 2.

**Table 4:** Mineral content in the feces and urine of growing–finishing pigs.

Treatment ^1^	P	Ca	Mg	Cu	Zn	Fe	P	Ca	Mg	Cu	Zn	Fe
	Grower						Finisher					
Feces	g/kg DM	g/kg DM	g/kg DM	mg/kg DM	mg/kg DM	mg/kg DM	g/kg DM	g/kg DM	g/kg DM	mg/kg DM	mg/kg DM	mg/kg DM
NC ^2^	4.64 ^b^	5.31 ^b^	2.41 ^b^	14.05	105.9 ^a^	91.60	3.40 ^b^	3.80 ^ab^	2.09 ^b^	8.23	56.86 ^a^	61.54
250 ^3^	4.47 ^bc^	5.22 ^b^	2.18 ^bc^	13.96	102.7 ^a^	90.76	3.21 ^c^	3.61 ^c^	1.93 ^b^	8.19	56.21 ^ab^	61.49
500 ^3^	4.30 ^cd^	5.20 ^b^	2.27 ^bc^	13.81	95.75 ^b^	90.01	3.18 ^c^	3.67 ^bc^	1.98 ^b^	8.16	54.59 ^ab^	62.11
1000 ^3^	4.08 ^d^	5.16 ^b^	2.25 ^bc^	13.61	91.91 ^bc^	89.31	3.07 ^c^	3.62 ^bc^	1.78 ^c^	8.15	53.63 ^b^	62.10
1500 ^3^	4.02 ^d^	5.18 ^b^	2.00 ^c^	13.38	89.04 ^c^	89.11	3.00 ^c^	3.82 ^ab^	1.83 ^c^	8.11	53.05 ^b^	62.43
PC ^4^	5.05 ^a^	5.71 ^a^	2.31 ^a^	14.06	106.2 ^a^	91.45	3.68 ^a^	3.95 ^a^	2.31 ^a^	8.21	56.95 ^a^	61.61
SEM ^5^	0.057	0.032	0.052	0.071	1.07	0.513	0.033	0.025	0.029	0.041	0.371	0.501
*p*-value												
TRT ^6^	<0.001	<0.001	<0.001	0.295	<0.001	0.632	<0.001	<0.001	<0.001	0.101	0.001	0.994
PHY ^7^	<0.001	0.097	<0.001	0.337	<0.001	0.632	<0.001	0.001	<0.001	0.966	0.005	0.984
Linear ^8^	<0.001	0.016	0.002	0.049	<0.001	0.123	<0.001	0.682	0.149	0.463	<0.001	0.573
Quadratic ^8^	0.220	0.192	<0.001	0.698	0.390	0.785	0.003	<0.001	0.002	0.976	0.778	0.982
Urine	mmol/L	mmol/L	mmol/L	µmol/L	µmol/L	µmol/L	mmol/L	mmol/L	mmol/L	µmol/L	µmol/L	µmol/L
NC ^2^	1.46 ^ab^	1.11 ^a^	0.924	0.511 ^a^	3.87 ^c^	4.55 ^bc^	1.16 ^b^	1.19 ^a^	0.953 ^b^	0.633 ^a^	5.69	6.52 ^a^
250 ^3^	1.35 ^b^	1.10 ^a^	0.859	0.501 ^b^	4.08 ^b^	4.35 ^bc^	1.10 ^c^	1.18 ^a^	0.950 ^b^	0.618 ^abc^	5.70	6.33 ^a^
500 ^3^	1.35 ^b^	0.844 ^b^	0.842	0.487 ^b^	5.03 ^a^	5.63 ^a^	1.19 ^b^	1.13 ^ab^	0.943 ^b^	0.607 ^bc^	5.49	6.33 ^a^
1000 ^3^	1.23 ^bc^	0.710 ^bc^	0.886	0.474 ^bc^	4.83 ^a^	5.69 ^a^	0.959 ^c^	1.04 ^b^	1.03 ^a^	0.592 ^c^	5.42	6.07 ^a^
1500 ^3^	1.17 ^c^	0.655 ^c^	0.869	0.411 ^d^	4.68 ^a^	4.25 ^c^	0.852 ^d^	0.946 ^b^	1.04 ^a^	0.509 ^d^	4.86	5.70 ^b^
PC ^4^	1.58 ^a^	1.10 ^a^	0.936	0.520 ^a^	3.88 ^c^	4.67 ^b^	1.46 ^a^	1.21 ^a^	0.961 ^b^	0.638 ^a^	5.79	6.53 ^a^
SEM ^5^	0.035	0.048	0.015	0.009	0.023	0.144	0.047	0.025	0.010	0.011	0.083	0.077
*p*-value												
TRT ^6^	<0.001	<0.001	0.422	<0.001	<0.001	<0.001	<0.001	<0.001	<0.001	<0.001	0.001	<0.001
PHY ^7^	0.002	<0.001	0.647	<0.001	0.002	<0.001	<0.001	0.001	<0.001	<0.001	0.002	0.001
Linear ^8^	<0.001	<0.001	0.517	<0.001	0.001	0.008	<0.001	<0.001	<0.001	<0.001	<0.001	<0.001
Quadratic ^8^	0.931	0.750	0.307	0.001	0.013	<0.001	<0.001	0.080	0.058	<0.001	0.032	0.115

Note: See Table 2.

## Data Availability

The data that support the findings of this study are available from the corresponding author (W.S.), upon reasonable request.

## Supplementary Materials

The following supporting information can be downloaded at: https://www.mdpi.com/article/10.3390/ani12101294/s1, Table S1: Correlations between content of minerals in the chyme and their content in the blood, femur, faeces and urine; Table S2: Correlations between content of minerals in the blood and their content in the femur, faeces and urine; Table S3: Correlations between content of minerals in the femur and their content in the faeces and urine.

## References

[1] Dersjant-Li Y., Awati A., Schulze H., Partridge G. (2015). Phytase in non-ruminant animal nutrition: A critical review on phytase activities in the gastrointestinal tract and influencing factors. J. Sci. Food Agric..

[2] Tsai T.C., Dove R., Bedford M.R., Azain M.J. (2020). Effect of phytase on phosphorous balance in 20-kg barrows fed low or adequate phosphorous diets. Anim. Nutr..

[3] Humer E., Schwarz C., Schedle K. (2015). Phytate in pig and poultry nutrition. J. Anim. Physiol. Anim. Nutr..

[4] Létourneau-Montminy M.P., Narcy A., Lescoat P., Bernier J.F., Magnin M., Sauvant D., Jondreville C., Pomar C. (2011). Modeling the fate of dietary phosphorus in the digestive tract of growing pigs. J. Anim. Sci..

[5] Chu G.M., Komori M., Hattori R., Matsui T. (2009). Dietary phytase increases the true absorption and endogenous fecal excretion of zinc in growing pigs given a corn-soybean meal based diet. Anim. Sci. J..

[6] Peter C.M., Parr T.M., Parr E.N., Webel D.M., Baker D.H. (2001). The effects of phytase on growth performance, carcass characteristics, and bone mineralization of late-finishing pigs fed maize-soyabean meal diets containing no supplemental phosphorus, zinc, copper and manganese. Anim. Feed Sci. Technol..

[7] Grela E.R., Kumek R. (2002). Effect of feed supplementation with phytase and formic acid on piglet performance and composition of sow colostrum and milk. Vet. Med..

[8] Czech A., Grela E.R. (2004). Biochemical and haematological blood parameters of sows during pregnancy and lactation fed the diet with different source and activity of phytase. Anim. Feed Sci. Technol..

[9] Grela E.R., Muszyński S., Czech A., Donaldson J., Stanisławski P., Kapica M., Brezvyn O., Muzyka V., Kotsyumbas I., Tomaszewska E. (2020). Influence of phytase supplementation at increasing doses from 0 to 1500 FTU/kg on growth performance, nutrient digestibility, and bone status in grower-finisher pigs fed phosphorus-deficient diets. Animals.

[10] Hong B., Kim B.G. (2021). Supplemental phytase increases phosphorus digestibility in pigs regardless of phytase source or feed pelleting. Anim. Feed Sci. Technol..

[11] Silversides G., Scott T.A., Bedford M.R. (2004). The effect of phytase enzyme and level on nutrient extraction by broilers. Poult. Sci..

[12] Augspurger N.R., Webel D.M., Lei X.G., Baker D.H. (2003). Efficacy of an E. coli phytase expressed in yeast for releasing phytate-bound phosphorus in young chicks and pigs. J. Anim. Sci..

[13] NRC (2012). Nutrient Requirements of Swine.

[14] AOAC International (2016). Official Methods of Analysis of AOAC.

[15] Engelen A.J., van der Heeft F.C., Randsdorp P.H., Somers W.A., Schaefer J., van der Vat B.J. (2001). Determination of phytase activity in feed by a colorimetric enzymatic method: Collaborative interlaboratory study. J. AOAC Int..

[16] Crenshaw T.D., Peo E.R., Lewis A.J., Moser B.D., Olson D.G. (1981). Influence of age, sex and calcium and phosphorus levels on the mechanical properties of various bones in swine. J. Anim. Sci..

[17] Pointillart A., Guéguen L. (1993). Meal-feeding and phosphorus ingestion influence calcium bioavailability evaluated by calcium balance and bone breaking strength in pigs. Bone Miner..

[18] Shaw D.T., Rozeboom D.W., Hill G.M., Orth M.W., Rosenstein D.S., Link J.E. (2006). Impact of supplement withdrawal and wheat middling inclusion on bone metabolism, bone strength, and the incidence of bone fractures occurring at slaughter in pigs. J. Anim. Sci..

[19] Storskrubb A., Sevón-Aimonen M.L., Uimari P. (2010). Genetic parameters for bone strength, osteochondrosis and meat percentage in Finnish Landrace and Yorkshire pigs. Animal.

[20] Saraiva A., Donzele J.L., Oliveira R.F.M., Abreu M.L.T., Silva F.C.O., Guimarães S.E.F., Kim S.W. (2012). Phosphorus requirements for 60- to 100-kg pigs selected for high lean deposition under different thermal environments. J. Anim. Sci..

[21] Santos T.T., Walk C.L., Wilcock P., Cordero G., Chewning J. (2014). Performance and bone characteristics of growing pigs fed diets marginally deficient in available phosphorus and a novel microbial phytase. Can. J. Anim. Sci..

[22] Teixeira A.O., Anderson Corassa A., Moreira L.M., Nogueira E.T., Lopes J.B., Rocha C.M., Ferreira V.P.A. (2016). Bone characteristics of pigs fed different sources of phosphorus. Rev. Colom. Cienc. Pecua..

[23] Arredondo M.A., Casas G.A., Stein H.H. (2019). Increasing levels of microbial phytase increases the digestibility of energy and minerals in diets fed to pigs. Anim. Feed Sci. Technol..

[24] Madrid J., Martínez S., López C., Hernández F. (2013). Effect of phytase on nutrient digestibility, mineral utilization and performance in growing pigs. Livest. Sci..

[25] Varley P.F., Callan J.J., O’Doherty J.V. (2011). Effect of dietary phosphorus and calcium level and phytase addition on performance, bone parameters, apparent nutrient digestibility, mineral and nitrogen utilization of weaner pigs and the subsequent effect on finisher pig bone parameters. Anim. Feed Sci. Technol..

[26] Lei X.G., Ku P.K., Miller E.R., Yokoyama M.T. (1993). Supplementing corn-soybean meal diets with microbial phytase linearly improves phytate phosphorus utilization by weanling pigs. J. Anim. Sci..

[27] Gentile J.M., Roneker K.R., Crowe S.E., Pond W.G., Lei X.G. (2003). Effectiveness of an experimental consensus phytase in improving dietary phytate phosphorus utilization by weanling pigs. J. Anim. Sci..

[28] Jendza J.A., Dilger R.N., Adedokun S.A., Sands J.S., Adeola O. (2005). *Escherichia coli* phytase improves growth performance of starter, grower, and finisher pigs fed phosphorus-deficient diets. J. Anim. Sci..

[29] Rosenfelder-Kuon P., Siegert W., Rodehutscord M. (2019). Effect of microbial phytase supplementation on P digestibility in pigs: A meta-analysis. Arch. Anim. Nutr..

[30] Sung J.Y., Kim B.G. (2019). Prediction models for apparent and standardized total tract digestible phosphorus in swine diets. Anim. Feed Sci. Technol..

[31] González-Vega J., Stein H. (2014). Invited review—Calcium digestibility and metabolism in pigs. Anim. Biosci..

[32] Stein H.H., Boersma M.G., Pedersen C. (2006). Apparent and true total tract digestibility of phosphorus in field peas (*Pisum sativum* L.) by growing pigs. Can. J. Anim. Sci..

[33] Stein H.H., Adeola O., Cromwell G.L., Kim S.W., Mahan D.C., Miller P.S. (2011). Concentration of dietary calcium supplied by calcium carbonate does not affect the apparent total tract digestibility of calcium, but reduces digestibility of phosphorus by growing pigs. J. Anim. Sci..

[34] Almeida F.N., Stein H.H. (2010). Performance and phosphorus balance of pigs fed diets formulated on the basis of values for standardized total tract digestibility of phosphorus. Can. J. Anim. Sci..

[35] González-Vega J.C., Walk C.L., Liu Y., Stein H.H. (2014). The site of net absorption of Ca from the intestinal tract of growing pigs and effect of phytic acid, Ca level and Ca source on Ca digestibility. Arch. Anim. Nutr..

[36] Selle P.H., Ravindran V. (2008). Phytate-degrading enzymes in pig nutrition. Livest. Sci..

[37] Zeng Z.K., Li Q.Y., Zhao P.F., Xu X., Tian Q.Y., Wang H.L., Pan L., Yu S., Piao X.S. (2016). A new Buttiauxella phytase continuously hydrolyzes phytate and improves amino acid digestibility and mineral balance in growing pigs fed phosphorous-deficient diet. J. Anim. Sci..

[38] She Y., Liu Y., González-Vega J.C., Stein H.H. (2018). Effects of graded levels of an *Escherichia coli* phytase on growth performance, apparent total tract digestibility of phosphorus, and on bone parameters of weanling pigs fed phosphorus-deficient corn-soybean meal based diets. Anim. Feed Sci. Technol..

[39] Adeola O. (1995). Digestive utilization of minerals by weanling pigs fed copper-and phytase-supplemented diets. Can. J. Anim. Sci..

[40] Bikker P., Jongbloed A.W., Thissen J.T.N.M. (2012). Meta-analysis of effects of microbial phytase on digestibility and bioavailability of copper and zinc in growing pigs. J. Anim. Sci..

[41] Espinosa C.D., Stein H.H. (2021). Digestibility and metabolism of copper in diets for pigs and influence of dietary copper on growth performance, intestinal health, and overall immune status: A review. J. Anim. Sci. Biotechnol..

[42] Collins J.F., Prohaska J.R., Knutson M.D. (2010). Metabolic crossroads of iron and copper. Nutr. Rev..

[43] Jiao L.F., Zhang Q.H., Wu H., Wang C.C., Cao S.T., Feng J., Hu C.H. (2018). Influences of copper/zinc-loaded montmorillonite on growth performance, mineral retention, intestinal morphology, mucosa antioxidant capacity, and cytokine contents in weaned piglets. Biol. Trace Elem. Res..

[44] O’Doherty J.V., Gahan D.A., O’Shea C., Callan J.J., Pierce K.M. (2010). Effects of phytase and 25-hydroxyvitamin D_3_ inclusions on the performance, mineral balance and bone parameters of grower-finisher pigs fed low-phosphorus diets. Animal.

[45] Klem T.B., Bleken E., Morberg H., Thoresen S.I., Framstad T. (2010). Hematologic and biochemical reference intervals for Norwegian crossbreed grower pigs. Vet. Clin. Path..

[46] Petersen G.I., Pedersen C., Lindemann M.D., Stein H.H. (2011). Relative bioavailability of phosphorus in inorganic phosphorus sources fed to growing pigs. J. Anim. Sci..

[47] Haeney R.P., Abrams S., Dawsonhughes B., Looker A., Marcus R., Matkovic V., Weaver C. (2000). Peak Bone Mass. Osteoporos. Int..

[48] Calvo M.S., Park Y.K. (1996). Changing phosphorus content of the U.S. Diet: Potential for adverse effects on bone. J. Nutr..

[49] Cashman K.D. (2007). Diet, nutrition, and bone health. J. Nutr..

[50] Revy P.S., Jondreville C., Dourmad J.Y., Nys Y. (2004). Effect of zinc supplemented as either an organic or an inorganic source and of microbial phytase on zinc and other minerals utilisation by weanling pigs. Anim. Feed Sci. Technol..

[51] Adedokun S.A., Owusu-Asiedu A., Ragland D., Plumstead P., Adeola O. (2015). The efficacy of a new 6-phytase obtained from *Buttiauxella* spp. expressed in *Trichoderma reesei* on digestibility of amino acids, energy, and nutrients in pigs fed a diet based on corn, soybean meal, wheat middlings, and corn distillers’ dried grains with solubles. J. Anim. Sci..

[52] McCormick K., Walk C.L., Wyatt C.L., Adeola O. (2017). Phosphorus utilization response of pigs and broiler chickens to diets supplemented with antimicrobials and phytase. Anim. Nutr..

[53] Jendza J.A., Adeola O. (2009). Water-soluble phosphorus excretion in pigs fed diets supplemented with microbial phytase. Anim. Sci. J..

[54] De Baaij J.H., Hoenderop J.G., Bindels R.J. (2015). Magnesium in man: Implications for health and disease. Physiol. Rev..

[55] Woyengo T.A., Cowieson A.J., Adeola O., Nyachoti C.M. (2009). Ileal digestibility and endogenous flow of minerals and amino acids: Responses to dietary phytic acid in piglets. Br. J. Nutr..

[56] Wu G. (2018). Principles of Animal Nutrition.

