# Supplementary material for: Effect of Microbial Phytase on Ileal Digestibility of Minerals, Plasma and Urine Metabolites, and Bone Mineral Concentrations in Growing–Finishing Pigs
Source: Animals (Basel). 2022 May 18;12(10):1294. doi: 10.3390/ani12101294 (PMC9137887; doi:10.3390/ani12101294)
Supplement: Supplementary file 1 [file animals-12-01294-s001.zip › animals-1684256-supplementary.pdf]

Effect of microbial phytase on ileal digestibility of minerals, plasma and urine metabolites, and bone mineral concentrations in growing-finishing pigs

Anna Czech, Wioletta Samolińska, Ewa Tomaszewska, Siemowit Muszyński, Eugeniusz R. Grela

Supplementary materials

Table S1. Correlations between content of minerals in the chyme and their content in the blood, femur, faeces and urine.

Treatment [1]		NC [2]	250 [3]	500 [3]	1000 [3]	1500 [3]	PC [4]	NC [2]	250 [3]	500 [3]	1000 [3]	1500 [3]	PC [4]
		Grower						Finisher					
Chyme /blood	P	-0.467	0.612	-0.578	**-0.789**	**0.919**	**-0.987**	-0.515	**-0.934**	**0.982**	-0.688	0.571	**-1.000**
	Ca	0.099	**0.858**	0.126	0.538	**0.995**	-0.342	-0.668	**0.996**	-0.194	-0.051	-0.485	0.198
	Mg	-0.761	-0.237	**-0.990**	-0.020	-0.557	**-0.857**	-0.277	-0.069	-0.371	**0.945**	**-0.971**	0.511
	Cu	**0.918**	0.409	0.266	0.305	-0.713	**-0.903**	-0.742	-0.263	**-0.890**	0.092	-0.577	0.411
	Zn	0.256	0.507	**0.999**	**-0.986**	0.404	**0.983**	0.302	**-0.967**	-0.791	0.106	-0.170	0.062
	Fe	0.088	**0.855**	-0.445	**0.820**	-0.733	-0.579	-0.603	0.364	**0.959**	**0.984**	**0.978**	0.638
Chyme /	P	**-0.965**	0.265	0.488	-0.486	0.256	0.777	**0.961**	**0.946**	-0.416	0.695	-0.047	**0.968**

femur	Ca	-0.178	**0.965**	-0.236	**0.930**	0.001	0.230	0.170	**0.959**	-0.394	**0.878**	**-0.862**	**-0.897**
	Mg	-0.118	-0.888	-0.235	0.747	**-1.000**	0.407	0.771	0.808	0.090	**0.913**	**-0.983**	0.776
	Cu	**0.903**	**-1.000**	-0.657	**0.908**	-0.223	-0.794	0.589	**-0.892**	**0.982**	-0.033	-0.158	**-0.993**
	Zn	-0.191	0.560	0.274	**0.729**	**-0.963**	-0.420	**-0.876**	-0.604	0.699	-0.999	**-0.882**	**0.998**
	Fe	-0.668	0.320	-0.425	0.388	-0.507	-0.707	**1.000**	**-0.877**	-0.717	**0.993**	-0.272	**-0.891**
Chyme / faeces	P	0.386	0.247	-0.321	-0.109	0.587	0.175	0.056	**-0.918**	-0.288	**0.825**	**1.000**	-0.690
	Ca	-0.488	-0.059	0.663	-0.637	**0.956**	-0.394	**-0.848**	**0.762**	-0.587	**0.814**	**0.983**	**-0.843**
	Mg	-0.352	-0.159	-0.677	0.680	0.174	-0.305	0.431	**0.995**	**0.955**	-0.420	-0.769	-0.087
	Cu	0.760	**0.851**	0.026	0.325	0.266	0.057	**0.934**	0.149	-0.058	-0.139	**-0.942**	0.576
	Zn	0.038	0.606	**0.992**	**0.999**	-0.575	**0.814**	**-0.922**	**0.952**	-0.674	-0.373	**0.966**	-0.562
	Fe	0.441	**-0.847**	0.433	0.352	-0.615	**0.997**	-0.384	0.408	**0.957**	-0.174	-0.486	-0.232
Chyme / urine	P	0.771	**0.995**	-0.078	-0.123	**0.951**	0.276	0.134	-0.153	0.384	0.439	**0.971**	**1.000**
	Ca	**0.962**	0.175	**-0.967**	**-0.801**	-0.777	**0.819**	-0.039	**1.000**	0.247	**-0.835**	**0.851**	**0.978**
	Mg	0.732	0.282	**0.909**	0.387	0.637	**0.946**	0.863	**0.998**	-0.372	**0.999**	-0.729	**-0.999**
	Cu	**0.878**	-0.556	0.748	-0.479	-0.006	-0.444	-0.124	0.632	-0.366	-0.129	**-0.908**	-0.667

Zn	-0.503	**-0.999**	0.196	**0.862**	**0.998**	**0.940**	-0.529	-0.213	**0.990**	-0.139	-0.671	0.468
Fe	-0.246	**0.991**	**-1.000**	-0.069	0.315	**-0.876**	0.533	-0.266	**0.964**	**0.750**	0.367	0.055

Color-coded correlation matrix. Bold values indicate significant correlations ($P < 0.05$); [1] Diets 1–5 were P-deficient diets with 0 (NC), 250, 500, 1000, or 1500 phytase units FTU/kg, respectively. A sixth diet (positive control; PC) was formulated with increased dicalcium phosphate to meet the nutrient requirements of the pigs; [2] NC – negative control; [3] Phytase level added to NC; [4] PC – positive control.

Table S2. Correlations between content of minerals in the blood and their content in the femur, faeces and urine.

Treatment [1]		NC [2]	250 [3]	500 [3]	1000 [3]	1500 [3]	PC [4]	NC [2]	250 [3]	500 [3]	1000 [3]	1500 [3]	PC [4]
		grower						finisher					
Blood / femur	P	0.684	-0.601	0.430	-0.154	-0.145	-0.868	-0.733	**-0.999**	-0.236	**-1.000**	**-0.847**	**-0.971**
	Ca	**0.962**	**0.963**	**0.930**	0.811	0.106	**-0.993**	0.619	**0.928**	**0.978**	0.434	**0.862**	-0.611
	Mg	0.233	-0.742	**0.929**	0.649	0.564	0.025	**-0.825**	0.781	**0.891**	0.729	**0.998**	**0.939**
	Cu	0.658	-0.402	-0.236	-0.122	0.843	-0.891	**-0.979**	-0.201	**-0.789**	**-0.998**	**0.898**	-0.300
	Zn	**0.900**	-0.430	0.627	-0.833	-0.637	-0.496	-0.725	0.381	-0.115	-0.055	-0.316	0.126
	Fe	0.683	0.765	**0.984**	0.845	-0.215	**0.970**	-0.602	-0.767	**-0.885**	**0.956**	-0.468	**-0.918**
Blood / faeces	P	0.741	0.147	-0.588	-0.525	**0.859**	-0.016	-0.885	0.715	-0.101	**-0.978**	0.596	0.700
	Ca	**0.987**	-0.500	**0.826**	**-0.992**	**0.920**	**0.998**	0.173	0.698	**0.908**	0.539	-0.313	-0.695
	Mg	0.220	**0.999**	**0.774**	0.719	**-0.914**	0.752	0.747	-0.167	-0.080	-0.694	**0.900**	**-0.901**
	Cu	**0.990**	**0.999**	**0.971**	**-0.802**	**-0.866**	0.378	-0.454	**0.915**	-0.404	**-0.999**	**0.817**	**0.982**
	Zn	-0.614	0.711	**0.997**	**-0.976**	**-0.981**	**0.906**	0.091	**-0.999**	**0.985**	**-0.962**	0.091	0.790
	Fe	0.455	0.757	0.615	-0.247	**0.987**	-0.645	-0.504	-0.701	**0.835**	0.006	-0.292	**-0.897**

	P	**-0.923**	0.533	0.859	0.707	**0.996**	-0.119	**-0.918**	-0.210	0.551	**-0.954**	0.360	**-1.000**
	Ca	-0.178	0.656	-0.376	0.075	-0.707	0.258	0.769	**0.995**	**0.903**	-0.506	**-0.872**	0.397
Blood /	Mg	0.648	-0.800	-0.841	**-0.930**	0.284	-0.643	0.245	-0.010	**1.000**	**0.947**	**0.872**	-0.509
urine	Cu	0.617	**-0.986**	**0.839**	**-0.978**	0.706	0.786	-0.573	**-0.914**	0.750	**0.975**	**0.866**	**0.405**
	Zn	**-0.964**	-0.484	0.246	**-0.934**	0.347	**0.986**	0.649	0.455	-0.698	**0.970**	-0.617	**-0.853**
	Fe	**-0.987**	**0.917**	0.426	0.627	0.415	0.115	0.353	**-0.995**	**0.850**	**0.857**	0.554	0.804

Note: See in table S1.

Table S3. Correlations between content of minerals in the femur and their content in the faeces and urine.

Treatment [1]		NC [2]	250 [3]	500 [3]	1000 [3]	1500 [3]	PC [4]	NC [2]	250 [3]	500 [3]	1000 [3]	1500 [3]	PC [4]
		Grower						Finisher					
Femur /faeces	P	0.017	0.702	**-0.983**	**0.922**	-0.632	-0.483	0.332	-0.739	**0.990**	**0.979**	-0.078	**-0.850**
	Ca	**0.993**	-0.248	-0.844	-0.876	-0.293	**-0.985**	-0.667	**0.915**	**0.975**	**0.993**	-0.752	**0.994**
	Mg	**-0.897**	-0.720	-0.845	**0.995**	-0.181	**0.935**	-0.242	0.485	0.381	-0.013	**0.873**	-0.695
	Cu	0.543	-0.367	**0.925**	0.691	**-0.999**	**-0.999**	0.261	-0.580	-0.243	**0.994**	0.479	-0.475
	Zn	**-0.897**	0.329	**-0.999**	0.692	0.775	-0.344	0.620	-0.332	0.058	0.325	**-0.974**	-0.508
	Fe	0.961	0.158	-0.721	-0.726	-0.368	0.889	-0.386	0.080	0.132	-0.288	-0.709	0.648
Femur / urine	P	**-0.912**	0.356	0.832	-0.807	-0.054	-0.390	0.404	0.176	0.680	**0.951**	0.192	**0.975**
	Ca	-0.441	0.428	**0.987**	-0.523	0.629	-0.370	**0.978**	**0.961**	0.794	**-0.997**	**-0.999**	**-0.969**
	Mg	-0.590	0.191	**0.987**	-0.324	-0.632	0.638	0.344	0.616	**0.890**	**0.910**	**0.842**	-0.774
	Cu	**0.999**	0.550	-0.700	-0.088	**0.976**	**-0.953**	0.729	-0.214	-0.186	**-0.987**	0.557	0.751
	Zn	-0.752	-0.582	**0.937**	**0.975**	**-0.944**	0.806	0.053	-0.650	0.791	0.190	**0.942**	0.411
	Fe	-0.557	0.443	**0.936**	**0.946**	**-0.978**	-0.439	0.534	0.696	**-0.998**	0.667	**-0.995**	-0.503

Note: See in table S1.
